# Inhibition of the BMP pathway prevents development of Barrett’s-associated adenocarcinoma in a surgical rat model

**DOI:** 10.1093/dote/doab072

**Published:** 2021-10-28

**Authors:** Wytske M Westra, Danielle Straub, Francesca Milano, Navtej S Buttar, Kenneth K Wang, Kausilia K Krishnadath

**Affiliations:** Center for Experimental and Molecular Medicine (CEMM), AUMC, Amsterdam, The Netherlands; Department of Gastroenterology and Hepatology, Amsterdam UMC, Amsterdam, The Netherlands; Department of Gastroenterology and Hepatology, Mayo Clinic, Rochester, Minnesota, USA; Center for Experimental and Molecular Medicine (CEMM), AUMC, Amsterdam, The Netherlands; Center for Experimental and Molecular Medicine (CEMM), AUMC, Amsterdam, The Netherlands; Department of Gastroenterology and Hepatology, Mayo Clinic, Rochester, Minnesota, USA; Department of Gastroenterology and Hepatology, Mayo Clinic, Rochester, Minnesota, USA; Department of Gastroenterology and Hepatology, University of Antwerp, Antwerp, Belgium; Laboratory of Experimental Medicine and Paediatrics, Antwerp, Belgium

**Keywords:** Gastro-Duodeno-Esophageal Reflux Disease, Barrett's Esophagus, Intestinal Metaplasia, Esophageal Adenocarcinoma, Animal Model, BMP4, Noggin

## Abstract

**Introduction:**

Esophageal adenocarcinoma (EAC) is an aggressive cancer, associated with reflux esophagitis and intestinal metaplasia (IM). One underlying biological mechanism, which possibly drives the development of EAC, is the dysregulated expression of Bone Morphogenetic Proteins (BMPs).

**Aim:**

To investigate if local delivery of Noggin, a BMP antagonist, reduced EAC.

**Methods:**

After obtaining proof of principal on local delivery of a Noggin/Sucralfate substance, a randomized controlled trial to test the effects of Noggin on EAC development was performed in a surgical rat model. In the model, an esophago-jejunostomy leads to development of reflux-esophagitis, IM and eventually EAC. Rats were treated by Noggin/Sucralfate or Sucralfate alone. Treatment was administered from 26 to 29 weeks after the operation.

**Results:**

Of the 112 operated rats, 52 survived beyond 26 weeks. Finally, 25 rats treated with Noggin/Sucralfate and 21 with Sucralfate, were evaluated. At the end, 39 (85%) of the animals had IM while 28 (61%) developed cancer. There were significantly more cancers in the Noggin/Sucralfate arm (50%) versus the Sucralfate group (73%) (Chi square, *P* < 0.05). Most cancers were mucous producing T3 adenocarcinomas. There were no significant differences in the amount of IM, size or grade of the cancers, or expression of columnar and squamous markers between the two groups.

**Conclusion:**

In this study, we demonstrated that inhibition of BMPs by Noggin reduced development of EAC in a surgical esophagitis-IM-EAC rat model. In future, effective targeting of the BMP pathway with selective BMP-inhibitors could become an important asset to improve EAC patient outcome.

## INTRODUCTION

Barrett’s esophagus (BE) is a condition caused by chronic gastro-duodeno-esophageal reflux (DGERD) in which the normal squamous mucosa of the distal esophagus is replaced by an intestinal type columnar epithelium, called intestinal metaplasia (IM).^1–4^ Patients with BE are at an increased risk for esophageal adenocarcinoma (EAC).^5–10^ EAC carries a poor prognosis, with an overall 5-year survival of less than 20%.^11^ In Western countries, the incidence of BE and is EAC is rising.^12–16^ Novel insight in the biological events giving rise to BE and EAC is of paramount importance to develop novel strategies for the prevention and treatment of EAC.

Our group previously demonstrated^17^^,^^18^ that BMP4, a member of the TGF- beta family, is one of the key factors for the development of esophageal (intestinal) metaplasia. BMPs have a major role during embryogenesis and in the homeostasis of tissues during adulthood. BMPs, together with Sonic Hedgehog (Shh), Notch and Wnts, are involved in transforming the primordial gut epithelium into an intestinal type of mucosa.^19^^,^^20^ Effects of BMPs are regulated by a range of natural inhibitors, which include Noggin, Chordin and Gremlin. Our studies showed that BMP4 is upregulated in esophagitis and (intestinal) metaplasia.^17^ We also demonstrated that the BMP4/pSMAD pathway is upregulated in the surgical rat esophagitis-IM model 20–22 weeks post esophago-jejunostomy and that BMP4 overexpression together with CDX2 induces upregulation of intestinal type of genes in a surgical mouse esophagitis-IM model.^17^^,^^21^ The role of BMPs and their antagonists in the development and progression of EAC is poorly understood. BMPs can either act as tumor suppressive or promote tumor growth. Their actions not only depend on the type of BMP and the type of cancer, but also on the stage of cancer development.^22^^,^^23^ For example, BMPs are highly expressed in ovarian cancer, and their expression has been shown to be inversely correlated to tumor differentiation and overall survival. BMP6 is expressed in esophageal squamous cell carcinoma and its expression is correlated with poor tumor differentiation and as a result worse outcome.^24^ Also, different BMPs are highly expressed in several types of gastrointestinal cancer including colorectal (BMP4/BMP7^25^) and hepatocellular cancer (BMP4/BMP7 and BMP9^26^).^27^ In diffuse type stomach cancer high levels of BMP2 and 4 have been associated with aggressive tumor behaviour, increasing epithelial mesenchymal transition and thus metastatic potential.^28^ Others have shown inhibition of BMP signaling, either by overexpression of GREM1 or as a result of SMAD4/BMPR1A mutations, to be a key event in several hereditary polyposis syndromes.^29^

We hypothesized that BMP signaling is important in the malignant transformation of Barrett’s esophagus and that inhibiting BMPs could be a target for tumor prevention and treatment. As one of the most well-known and potent natural antagonists of BMPs, Noggin has high affinity for BMP2, BMP4 and BMP7. Previous studies have shown Noggin to be capable of inhibiting the BMP pathway in vivo.^30^

A challenge is to demonstrate that Noggin can prevent cancer under the complex patho-physiological conditions as exist in DGERD as seen in Barrett’s patients. The best available physiological IM-EAC animal model is the surgical rat model^31^^,^^32^ In the IM-EAC rat model the complete cascade of reflux, injury of the esophageal mucosa by reflux of bile and acids followed by repair and replacement of the normal squamous mucosa by intestinal metaplasia (IM) and eventually progression to EAC is represented.^33^^,^^34^ The current study was designed to investigate whether in vivo inhibition of the BMP pathway could prevent formation of IM associated EAC in a surgical rat model.

## MATERIALS AND METHODS

### In vitro testing of noggin/Sucralfate in cancer cell cultures

The colon cancer, Ht29 cells (ATCC, Molsheim Cedex, France) were maintained in a humidified atmosphere containing 5% co_2_ at 37°C as described previously by Milano et al.^17^ After 2–3 weeks of culturing, cells were incubated with 5 μg/mL recombinant Noggin/Fc Chimera (R&D systems) and/or Sucralfate in different dosing combinations and time points.

### Western blot analysis

Preparation of the cells for and sodium dodecyl sulfate-polyacrylamide gel electrophoreses (SDS-PAGE) on the resulting cell lysate was performed as described previously by our group.^17^

### Antibodies

The antibodies that were used for the different procedures are described in Supplementary Table 1.

### The surgical rat esophagitis—IM—EAC model

The study was approved by the institutional animal ethical committee (DEC 101039). Six to eight-week-old male Sprague–Dawley rats were purchased from Harlan (Harlan Europe) and housed and fed 2–4 per cage under standard laboratory conditions. For induction of jejuno-esophageal reflux, a modified Levrat’s esophagojejunostomy was performed as previously described by dr. Buttar et al.^33^ (Supplementary Information 1 and Supplementary Table 2).

### Early effects of noggin in the surgical rat model

A proof of principle study was performed to evaluate if a Noggin/Sucralfate mixture decreases the BMP activity in the inflamed esophagus after oral administration. Sucralfate was used as a vehicle for oral delivery. A total of 20 male Sprague Dawley rats were included in the study. About, 15 rats underwent esophago-jejunostomy. After a period of four weeks, these rats were randomized into three groups receiving either: Noggin/Sucralfate (group 1, *n* = 5), Sucralfate only (group 2, *n* = 5), and no treatment (group 3, *n* = 5). Five rats were not operated and kept under control conditions to obtain normal tissues (group 4, *n* = 5). Recombinant Noggin was then administered twice daily via oral gavage, at a dose of 25 μg Noggin diluted in 75 μL Sucralfate (200 mg Sucralfate/ml) for at least 4 days. The rationale for the dosage is given in the Supplementary Information 2.

### In vivo effects of noggin on EAC development

To study the in vivo effects of Noggin on the development of EAC, a randomized controlled study was performed. In this study, 112 rats were operated. Rats that survived 26 weeks post-surgery were randomly divided into a Noggin/Sucralfate (Noggin) and Sucralfate only (Sucralfate) group. From previous reports it is known that at week 26 around 50% of the animals will have developed EAC and 90% will have EAC around week 29.^33^^,^^35^ Therefore, from week 26 after surgery the rats were administered Noggin/Sucralfate or Sucralfate only twice daily for a total duration of three weeks. Based on the pilot study Noggin was given at a dose of 25 μg diluted in 75 μL (=15 mg) sucralfate. All Noggin was supplied by R&D systems and was tested for efficacy prior to their use. (Supplementary Table 3).

### Autopsy and harvesting of tissues

At the end of the experimental period rats were euthanized and analyzed as follows: A midline incision was made from the laryngopharynx to the lower abdomen, the site of the anastomosis was identified and the esophagus cut at the level of the larynx and 2 mm above the anastomosis. The esophagus was then opened longitudinally and examined for presence of intestinal metaplasia, esophagitis or adenocarcinoma. The esophagus was fixed in formalin and then longitudinally divided into well oriented tissue slices.

### Histopathologic analysis

Histopathologic analysis was carried out on tissue sectioned into 4-μm slices, stained by hematoxylin & eosin. Diagnosis of IM was made based on the presence of intestinal type of columnar mucosa located in the esophagus, proximal to the jejuno-esophageal junction, and characterized by the presence of goblet cells positive for Alcian blue (ph 2.5) and PAS staining. Presence of dysplasia was assessed based on cell polarity, maturation, nuclear atypia and mitotic figures. Carcinoma was diagnosed in case severe dysplastic changes were seen and tumor cell invasion through the basement membrane was observed. The adenocarcinomas were classified based on differentiation grade and mucous production.

#### Inflammation scores

The degree of inflammation and reactive changes were scored on a scale of 0–4 for: hyperkeratosis, papillary hyperplasia, basal cell hyperplasia, presence of inflammatory cells in epithelium, lamina propria and submucosa. Ulcerations and/or erosions were scored as either 0 (absent) or 1(present) and a total inflammatory score was calculated for areas with or without ulcerations/erosions separately.

#### Immunohistochemistry

Tissue slides were processed as described previously.^17^ Esophageal and intestinal tissues were investigated for BMP pathway activity by immunohistochemistry (IHC) for pSMAD1,5,8 (pSMAD), indicating downstream signaling by BMPs. Down regulated PSMAD expression was scored calculating the percentage of negative nuclei in two sections of the esophagus: squamous epithelium next to the anastomosis and in the mid-esophagus. Immunohistochemistry for squamous (K5, K14, p63) and columnar markers (K8, PAS, MUC2 and CDX2) was performed according to previously described methods.^36^

### Sample size calculation

The primary endpoints were the number of EAC and secondary endpoints were the amount of intestinal metaplasia, the degree of inflammation and the expression of the BMP downstream target, pSMAD.

For the randomized study, group sample sizes of 30 were needed to achieve 80% power to detect a difference of 35 to 40% in EAC formation between the groups, using a Fisher’s Exact test, with a two-sided significance level of 0.05.

All further statistics and data management was performed using SPSS statistical software. Baseline categorical data was compared using the 2 × 2 test (or Fisher exact text when necessary because of small sample size). Baseline continuous data was compared using the Wilcoxon rank sum and Kruskal–Wallis tests. All tests are two sided, and a *P*-value <.05 was be considered statistically significant.

## RESULTS

### In vitro experiments

**Fig. 1:**
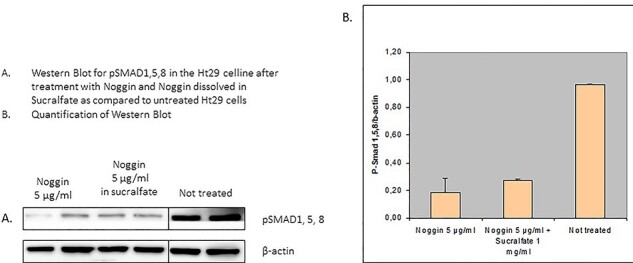
Ht29 cell line treated with Noggin (a BMP antagonist) in sucralfate.

To assess if Sucralfate could be used for Noggin delivery we first performed in vitro experiments. These experiments showed that Noggin inhibits the BMP pathway in Ht29 cells as demonstrated by a decrease in pSMAD. (Fig. 1) More importantly: Noggin and Sucralfate together gave equal inhibition of the BMP pathway as does Noggin alone, indicating that Sucralfate did not interfere with the function of Noggin (Fig. 1). From this study we concluded that Sucralfate was suitable as a vehicle for Noggin delivery in our in vivo experiments.

### Early effects of noggin in the surgical rat model

**Fig. 2:**
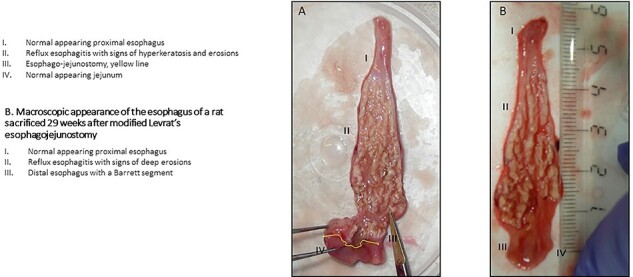
A. Macroscopic appearance of the esophagus of a rat sacrificed 6 weeks after the modified Levrat’s esophago-jejunostomy. B. Macroscopic appearance of the esophagus of a rat sacrificed 29 weeks after modified Levrat’s esophago-jejunostomy.

In this proof of principle study, we tested if the Noggin/Sucralfate mixture could be delivered orally at the anastomotic site in the esophagus of the model. 13 of 15 operated animals survived six weeks post-surgery. At sacrifice, 12 out of 13 surviving animals showed macroscopic signs of reflux esophagitis (Fig. 2B). There was no difference in macroscopic appearance between the operated treated and non-treated group.

#### Inflammation scores

**Fig. 3:**
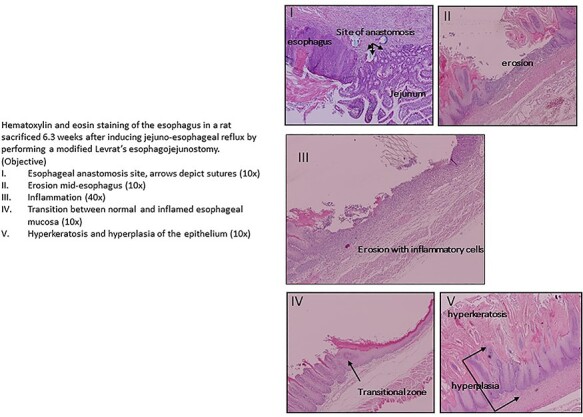
Inflammatory effects on the esophageal mucosa after surgical induction of jejuno-esophageal reflux.

An example of microscopic appearance of the inflamed esophagus is shown in Figure 3. Inflammation scores were lower in both of these groups when compared to the control animals: group 1 (Noggin in Sucralfate): 12 (11–13) and group 2 (Sucralfate): 9 (3–16) versus group 3 (control): 15(11–20) (average [range]), however these differences were not significant, most likely due to the small sample size. The combined Noggin and Sucralfate groups versus the non-treated operated rats showed a clear trend towards less inflammation with inflammation scores of 9.5 (3–13) versus 15 (11–20), *P* = 0.06. These results indicated that the Noggin/Sucralfate mixture could be effectively delivered through oral administration in the esophagus of this model.

#### Immunohistochemistry for BMP pathway activity

**Fig. 4:**
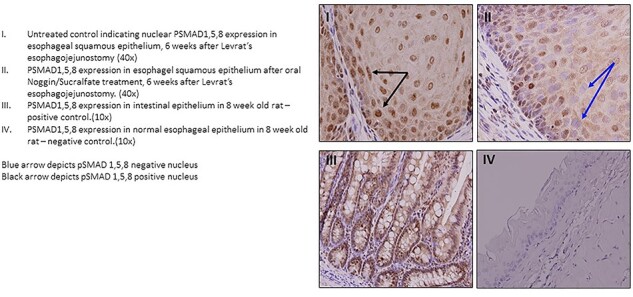
Immunohistochemistry for pSMAD1,5,8 expression six weeks after surgical induction of jejuno-esophageal reflux.

To assess the effect of Noggin on BMP pathway inhibition, the BMP pathway activity was evaluated by staining for its downstream target: pSMAD. By IHC we found that in group 1 (Noggin/Sucralfate) as compared to the non-treated group 40.7% ± 8.5 versus 17.5% ± 5.8 of nuclei were negative for pSMAD [*P* = 0.08, mean ± SEM] (Fig. 4). These results indicated that there was a trend towards decreased pSMAD activation in the Noggin-treated group, demonstrating that the oral local delivery of the Noggin/Sucralfate mixture was effective in inhibiting BMP activity.

### In vivo effects of noggin on EAC development

To evaluate the effects of Noggin on EAC development a randomized controlled trial for further testing of Noggin in the surgical model was performed. A flow chart of the study groups is presented in Supplementary Figure 1. Of the 112 operated rats, 60 rats did not survive beyond 26 weeks due to reaching pre-established humane endpoints (i.e. >25% decrease in bodyweight). These animals could not be included in the treatment phase of the trial and were euthanized at a median time of 68 days (range 0–177 days). Treatment was initiated at 26 weeks after surgery to investigate if the BMP inhibitor Noggin could attenuate cancer development. At 26 weeks post-surgery a total of 52 rats were randomized to Noggin/Sucralfate treatment (group 1, *n* = 27) or Sucralfate treatment only (group 2, *n* = 25).

During the treatment period another 6 rats (2/27, 7.4% in group 1 versus 4/25, 16% in group 2, *P* = 0.4) reached humane endpoints after an average of 5.7 days of treatment. Of the 46 rats that completed the three weeks of treatment, 25 were treated with Noggin in Sucralfate and 21 were treated with Sucralfate only.

There was no difference in the pre-operative weights between the groups at the start of the study. Animals in group 1 (Noggin/Sucralfate) weighed 310 ± 21 versus 319 ± 32 in group 2 (Sucralfate), (mean ± STDEV, gram). Animals in both groups lost equal amounts of weight after surgery, 13% in group 1 versus 14% in group 2. There was a significant difference in weight at sacrifice; 374 ± 33 (group 1) versus 404 ± 31 (group 2), *P* = 0.003. There was however no difference in amount of weight gained and/or loss during the treatment period: 3% in both treatment groups (Supplementary Figure 2).

#### Macroscopic findings at sacrifice

At 29 weeks, after the three weeks of Noggin or Sucralfate treatment, animals were euthanized, and tissues were harvested and processed as described. There was no significant difference in the location of the anastomosis between the two groups (16.9 ± 5.5 cm versus 16.0 ± 5.3 cm as measured from pylorus to anastomosis; mean ± SD). An example of macroscopic findings is shown in Figure 3B. The macroscopic appearance and average length of the IM segment in the Noggin-treated group was similar to that observed in the Sucralfate group (5.15 versus 4.60 mm, *P* = 0.5).

#### Inflammation scores

At sacrifice there were no differences in macroscopic length or severity of esophagitis when comparing Noggin to the sucralfate only group, also there was no significant difference in inflammation score (i.e. severity of inflammation) between both Noggin- and Sucralfate-treated groups: 12.5 (4–22) versus 11.2 (5–20), mean (range).

#### Immunohistochemistry for epithelial markers and downstream BMP targets

**Fig. 5:**
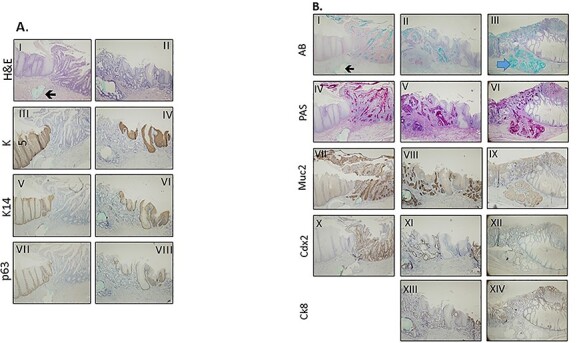
Squamous and columnar markers at site of surgical esophagojejunostomy.

Expression of squamous (K5, K14, p63) and columnar markers (K8, MUC2, CDX-2) indicate the development of IM with remnant squamous islands at the anastomotic site around week 16 after the operation (Fig. 5A). PAS and Alcian blue stainings indicate mucous producing goblet cells in IM and in invasive adenocarcinoma at different time points (Fig. 5).

#### Effects on intestinal metaplasia

**Fig. 6:**
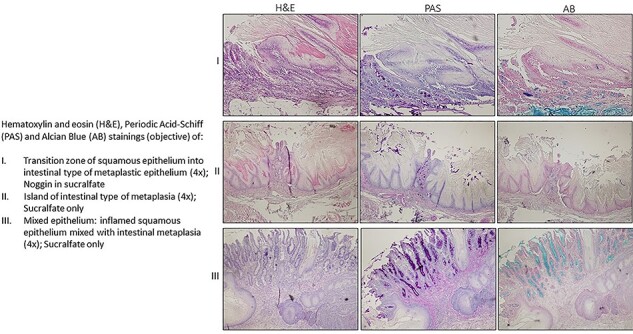
Microscopic appearance of esophagus 29 weeks after modified Levrat’s esophago-jejunostomy.

Based on previous studies at 26 weeks, intestinal type of metaplasia was expected to be found in approximately 100% in this model.^33^^,^^35^ To investigate if the Noggin treatment, which was initiated at week 26 effected the IM, the number of animals with IM and length of IM was determined. There was no significant difference in the number of animals with IM between the two groups. In the Noggin group 84% (21/25) had microscopic IM of any length versus 86% (18/21) in the Sucralfate group, Chi square test, *P* = 0.8. Also there was no significant difference in terms of length of the IM. 72% of animals (18/25) in the Noggin group versus 81% (17/21) in the Sucralfate group had IM > 1 mm, Chi square test, *P* = 0.5. Examples of microscopic findings are presented in Figure 6. Of interest is that we observed that the IM in the Noggin-treated group contained more islands and interspersed squamous epithelium (mixed type), (Fig. 6,III); however, the difference between the two groups was not significant (Chi square test, *P* = 0.187).

#### Effects on development of EAC

EAC development reaches its peak between 26–29 weeks in this model. At 29 weeks EAC was expected to be established in at least 80–90% of cases.^33^^,^^35^ There was a significant difference in the number of EAC between the two groups. At 29 weeks, in the Noggin-treated group 48% of animals (12/25) developed EAC as compared to the 76% (16/21) in the sucralfate group (Chi square test, *P* < 0.05). There was no significant difference in size of these cancers by student’s *t* test (Table 1). In both groups all but one of the tumors was of the mucous producing type. Most lesions were T3 tumors, corresponding to invasion of the adventitia. If EAC developed, there was no difference in T stadium between the Noggin-treated and Noggin/Sucralfate groups. Representative examples of the different types of dysplasia and cancers that the animals developed are shown in Supplementary Figure 3.

**Table 1:** 

Rat ID	Treatment group 1= Noggin/Sucralfate 2= Sucralfate only	BE segment > 1 mm 0=no 1=yes	Cyst 0=no 1=yes	EAC 0=no 1=yes	T stadium	Size EAC (mm) 999=Missing
1TT	1	0	0	0	0	
100	1	1	1	1	T1	0,25
4LT	1	1	1	1	T3	0,0625
400	1	1	0	0	0	
5RT	1	1	1	0	0	
5TT	1	1	1	1	T3	1
7LT	1	1	1	1	T3	3
7RT	1	1	0	0	0	
7TT	1	1	0	0	0	
700	1	1	1	1	T3	2,5
900	1	1	1	1	T3	2,5
11LT	1	1	1	1	T3	1
11RT	1	1	1	1	T3	0,25
11TT	1	1	0	0	0	
12LT	1	1	0	0	0	
12TT	1	1	0	0	0	
15LT	1	1	1	1	T3	3
15TT	1	1	1	1	T3	3
19LT	1	1	1	1	T3	6
19RT	1	1	1	1	T3	3,75
21TT	1	1	0	0	0	
2100	1	1	0	0	0	
2500	1	0	0	0	0	
30LT	1	0	0	0	0	
30RT	1	0	0	0	0	
2RT	2	0	1	0	0	
2TT	2	0	1	1	T3	4
3LT	2	1	1	1	T3	1,5
6TT	2	1	1	1	T3	1
600	2	1	0	1	T3	999
800	2	1	1	1	T2	3,75
10RT	2	1	1	1	T3	0,0625
10TT	2	1	1	1	T3	1,5
14TT	2	1	1	1	T3	2,5
1400	2	1	0	0	0	
16LT	2	1	1	1	T3	4
16RT	2	1	0	1	T1	999
16TT	2	1	0	0	0	
17RT	2	1	1	1	T3	1
22LT	2	1	0	0	0	
22RT	2	1	1	1	T3	3
2200	2	1	1	1	T3	5
23LT	2	1	0	0	0	
27TT	2	1	1	1	T3	4,5
29TT	2	0	1	1	T3	1,5
1300	2	1	0	1	T3	999

#### Effects on other gastrointestinal tissues

The proximal esophagus, jejunum, ileum and colon all showed normal macroscopic and histological appearance in both groups.

## DISCUSSION

Several BMPs have been associated with aggressive cancer phenotypes. BMPs have been found to be highly expressed in reflux esophagitis and IM and could be driving the malignant progression towards EAC. In this study we demonstrated for the first time that inhibition of BMPs by using Noggin prevented development of EAC in a jejuno-esophageal reflux disease-IM-EAC surgical rat model. The recombinant form of Noggin can be easily produced and its application could be translated towards the clinic. However, BMPs have important roles in the homeostasis of the normal intestinal type of epithelia as found in small bowel and colon and are essential for bone development and homeostasis. Systemic administration of Noggin could have unwanted side effects on these organs. We took advantage of the fact that BMPs exert their action in the extra-cellular space at the receptor level, and Noggin binds to BMPs to prevent receptor activation. By using a carrier substance to target the distal esophagus, Noggin could exert its action on the damaged esophageal mucosa in our model. The carrier substance Sucralfate (Aluminum Saccharose Sulfate) is a substance used as a mucosa protective in patients with esophageal inflammation.^37^^,^^38^,^38–40^ Sucralfate through binding with proteins adheres to damaged mucosal surfaces, as is seen in esophagitis, and functions as a barrier to prevent further damage by deleterious agents.^37^ Sucralfate is only minimally absorbed in the gut. Although the precise interaction between Noggin and Sucralfate has not been clarified, Sucralfate is known to have heparin like binding sides and for instance binds with low affinity to FGF.^41^^,^^42^ Noggin binds strongly to heparin *in vitro*, and to heparan sulfate proteoglycans on the surface of cells.^41^ Thus, theoretically, Sucralfate can bind to Noggin with relative lower affinity than Heparin and deliver it to the extra-cellular space and the cell surface. Once at the cell surface, Noggin can prevent BMP receptor activation through high affinity binding with the secreted BMPs, such as BMP-2, BMP4 and BMP-7.^43^ We confirmed in our in vitro experiments that the formula of Noggin/Sucralfate was as successful and effective in inhibiting the BMP/pSMAD pathway as Noggin alone.

Based on earlier research by Buttar et al. and Matsui et al.,^33^^,^^35^ up to 50% of animals will develop EAC around 26 weeks increasing to 90% around 29 weeks after the operation. We chose to treat the animals at 26 weeks post-surgery during the peak of EAC development. Unfortunately, the study was associated with a high dropout rate of animals. Most animals dropped out due to malnutrition either due to dysphagia caused by a peptic stricture or development of a malignant stenosis. This hampered the power of our study. Indeed those animals, which completed the study showed signs of development of cysts/EAC some even with local metastasis and/or signs of esophageal obstruction. A large number of animals also showed stasis of chow proximal of the anastomosis meaning a part of the orally administered Noggin may not have reached the distal esophagus. This may have confounded the results of the study and underestimated the effects of Noggin. In future studies, systemic treatment with more specific BMP targeting therapeutics could be more efficient for treatment of EAC.

Our secondary endpoint was to investigate the effect of Noggin on the IM that normally develops up to 100% of animals already 22 weeks after the operation. We observed an effect of oral Noggin treatment on BMP pathway activity and inflammation in our dosing study, which is before metaplastic changes develop in this model. In the randomized trial we started treatment at week 26 during which 90–100% of animals should have developed IM but also a large part may already have had developed EAC. Although we did not find a significant difference in terms of length of IM between the two groups, we did more often observed squamous island interspersed between the IM glands in the Noggin arm, suggesting that Noggin may have induced focal regression of the metaplastic lesions in the treated animals. For studying the preventive effect on development of IM an earlier time point for treatment of around 18–20 weeks and longer period of treatment would have been more appropriate.

The dosage of Noggin could have been a confounding factor. It is possible that a dose causing inhibition of BMP pathway activity in reflux esophagitis was not optimal to inhibit development of EAC and a higher dosage could have showed more profound effects. Finally, in the model the injury by bile and acidic reflux was ongoing also in the Noggin-treated rats, meaning there was a constant ‘conflict’ between factors that predispose to the development of reflux esophagitis, intestinal metaplasia and cancer and factors favoring inhibition of IM and EAC development.

In conclusion, this study shows that in this model local application of Noggin reduced the development of EAC. These findings warrant the use and development of more specific BMP inhibitors that could be tested in a more effective systemic setting for treatment of EAC.

## Disclosures and Grant Support

**WMW**: OESO young investigator award; Agiko scholarship ZonMw.

**KKW**: National Institutes of Health (NIH) U54 CA163004..

**KKK**: Koningin Wilhemina Fonds voor de Nederlandse Kankerbestrijding KWF-2010-4745, European research council ERC-StG 282079 TargetS4Barrett and ERC-POC 632258 BMP4EAC.

## Supplementary Material

Supplemental_Material_doab072

supplemental_figure_3_doab072
