# Supplementary material for: Inhibition of the BMP pathway prevents development of Barrett’s-associated adenocarcinoma in a surgical rat model
Source: Dis Esophagus. 2021 Oct 28;35(5):doab072. doi: 10.1093/dote/doab072 (PMC9113020; doi:10.1093/dote/doab072)
Supplement: Supplemental_Material_doab072 [file supplemental_material_doab072.docx]

**Supplemental Material**

**Supplementary Information 1 (SI 1) Establishing the surgical rat model**

General anaesthesia was induced and maintained using a mixture of xylazine (12mg/kg) and ketamine (75mg/kg). Animals were given baytrill 5 mg/kg s.c. as antibiotic prophylaxis and meloxicam 1 mg/kg s.c. and buprenorphine 0.02-0.06 mg/kg during surgery for pain management, both repeated postoperatively as needed. Subsequently, a midline laparatomy was performed, and the gastroesophageal junction was localized and mobilized while preserving vascular structures and the vagal nerve. The junction was ligated and transected and a 5 mm jejunostomy was made just distal to the ligament of Treitz using electrocautery. An end-to-side esophago-jejunostomy was created and the anastomosis was carefully placed between the two liver lobes. 1 cc of sterile 0.9% NaCl was left in the peritoneal cavity to compensate for blood loss. The abdominal wall was closed in two layers using 5-0 monofilament. Iron dextran (25 mg/kg) was then administered s.c. to prevent anaemia. Ensure (Abbott Nutrition) was provided once the rats were awake and chow was re-introduced during the second postoperative day. All equipment and medications used are summarized in Supplementary Table 2.

**Supplementary Information 2: Rationale dose Noggin and Sucralfate**

The dose was chosen based on earlier studies that used 500 ug/kg of Noggin for systemic (intraperitoneal/systemic) delivery of Noggin^37^, which would translate to an average of 200 µg per rat/day. Since we topically applied and targeted the Noggin to the anastomotic site by our carrier substance Sucralfate, we anticipated the minimally effective dosage to be at least 5-10x lower than a systemic dosage.

**Supplementary Table 1 (ST I): Antibodies used for different assays**

| Antibody | Technique | Company |
| --- | --- | --- |
| K5 | IHC | Epitomics, Burlingame, USA |
| K8 | IHC | Epitomics, Burlingame, USA |
| K14 | IHC | Abcam, Cambridge, UK |
| P63 | IHC | Santa Cruz Biotech, Texas, USA |
| CDX2 | IHC | Biogenex, Freemont, USA |
| MUC2 | IHC | Kind gift from Prof. Dr. J. Dekker, Erasmus MC, Rotterdam, The Netherlands |
| pSMAD1/5/8 | IHC | Millipore, Billerica, USA |
| pSMAD1/5/8 | WB | Cell signalling |
| βACTIN | WB | Santa Cruz Biotech, Texas, USA |

IHC = immunohistochemistry; WB = Western Blot

**Supplementary Table 3 (ST 2): Rat surgery, equipment and medication used**

| **Equipment, medication** | **Company** |
| --- | --- |
| Surgical instruments  Germinator 500 glass bead sterilizer | Fine Science Tools, Heidelberg, Germany  Stoelting Europe, Dublin, Ireland |
| Medications  * Baytril 2.5 %  * Iron Dextran  * Buprenorphine  * Ketamine  * Xylazine  * Meloxicam  * Sucralfate | Bayer, Mijdrecht, The Netherlands  Durvet, Blue Springs, USA  Shering-Plough, USA (now MSD)  AUV (now Covetrus), Cuijk, The Netherlands  AUV (now Covetrus), Cuijk, The Netherlands  Dopharma Veterinaire Farmaca,Raamsdonksveer, Nederland |
| Sutures  * 5.0 vicryl round circle  * 7.0 polypropylene 3/8 circle  * 4.0 softsilk, non absorbable | Ethicon (Johnson & Johnson), Amersfoort, The Netherlands  Ethicon (Johnson & Johnson), Amersfoort, The Netherlands  Covidien (now Medtronic), Watford, UK |
| Cage enrichment  * silicon bones (Dura Chew)  * rat tunnels | Bioserv, Flemington, USA  Bioserv, Flemington, USa |

**Supplementary Table 3 (ST 3): Noggin- recombinant mouse Noggin supplied by R&D systems**

| batch | Catalog number | Lot number | Concentration (mg/mL |
| --- | --- | --- | --- |
| I | 1967-NG/CF | ETY181009A | 0.245 |
| II | 1967-NG/CF | ETY191010A | 0.309 |
| III | 1967-NG/CF | ETY151010A | 0.601 |
| IV | 1967-NG/CF | ETY191010B | 0.309 |

**Supplementary Figure 1: Study Set up**


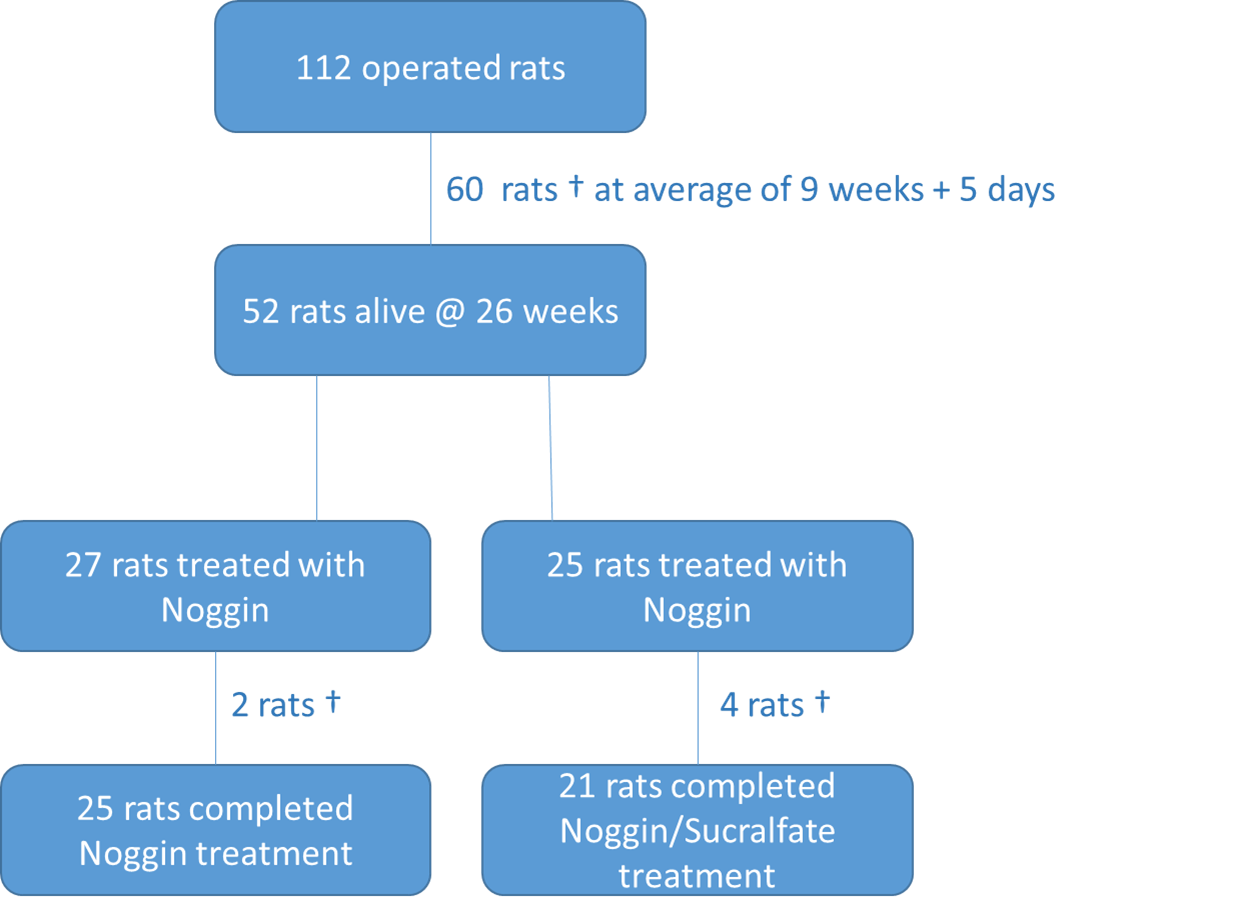


**Supplementary Figure 2: Weight of animals during study period**

**Supplementary Figure S3. Tumurous growth at the esophagojejunal anastomosis**

**
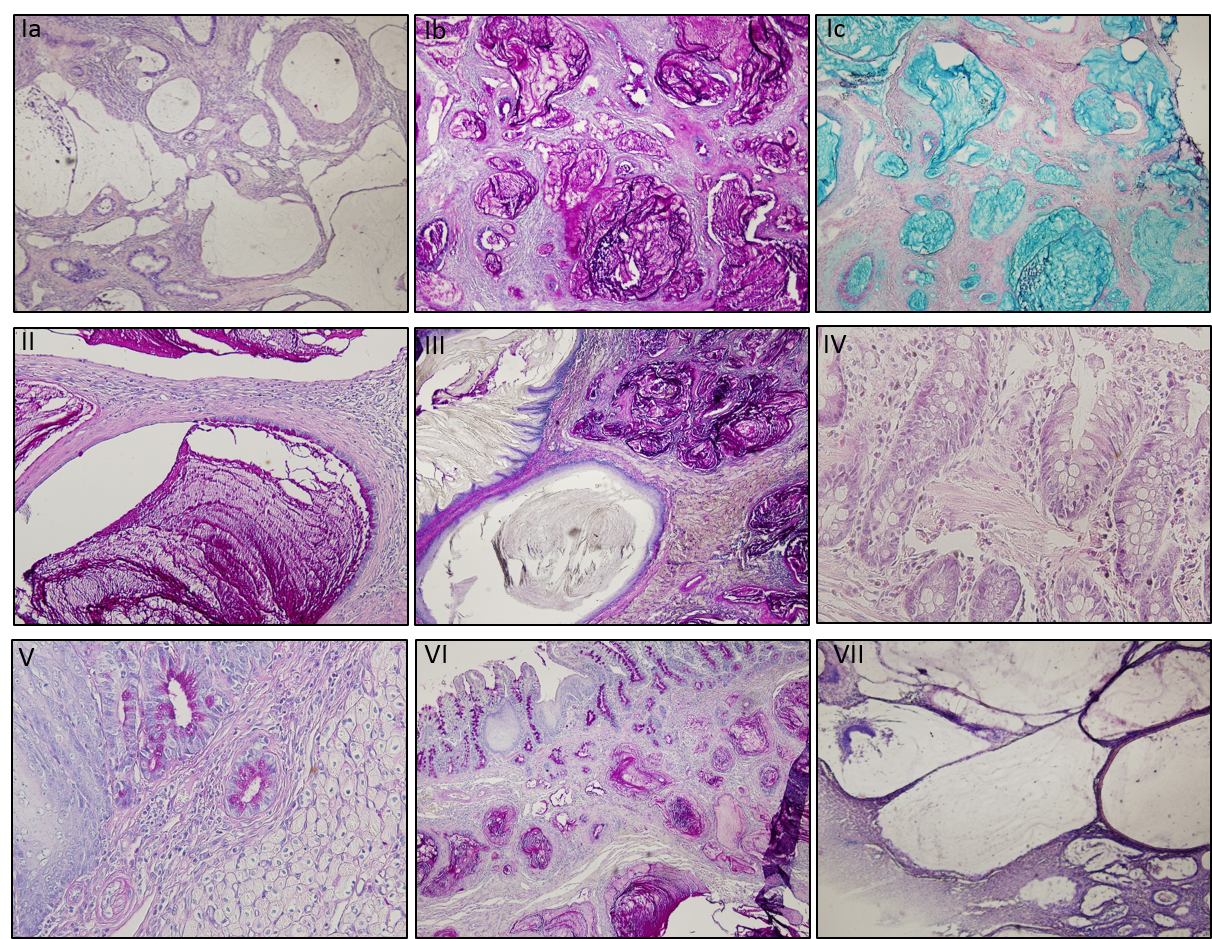

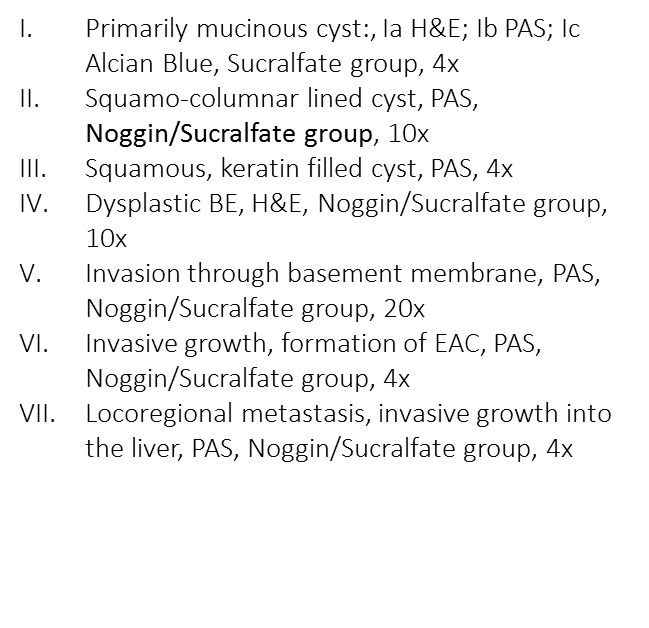
**
