# Supplementary figures and images for: Inhibition of the BMP pathway prevents development of Barrett’s-associated adenocarcinoma in a surgical rat model
Source: Dis Esophagus. 2021 Oct 28;35(5):doab072. doi: 10.1093/dote/doab072 (PMC9113020; doi:10.1093/dote/doab072)

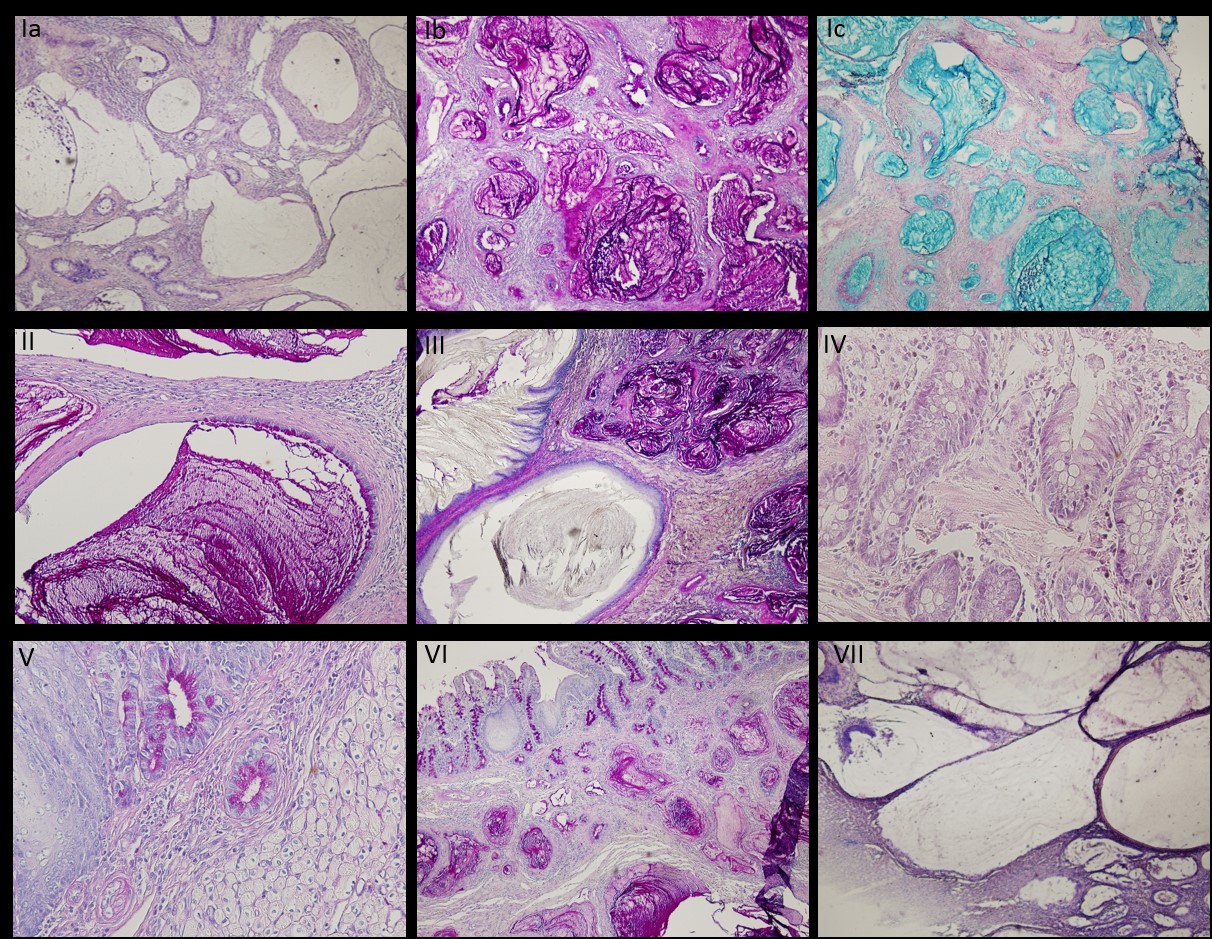

Supplement: supplemental_figure_3_doab072 [file supplemental_figure_3_doab072.jpeg]
